# The differences in crown formation during the splash on the thin water layers formed on the saturated soil surface and model surface

**DOI:** 10.1371/journal.pone.0181974

**Published:** 2017-07-27

**Authors:** Michał Beczek, Magdalena Ryżak, Agata Sochan, Rafał Mazur, Cezary Polakowski, Andrzej Bieganowski

**Affiliations:** Institute of Agrophysics PAS, Lublin, Poland; Centro de Investigacion Cientifica y de Educacion Superior de Ensenada Division de Fisica Aplicada, MEXICO

## Abstract

Splash is the first stage of a negative phenomenon–soil erosion. The aim of this work was to describe the crown formation quantitatively (as part of the splash erosion) and compare the course of this phenomenon on the thin water film formed on a smooth glass surface and on the surface of saturated soil. The height of the falling water drop was 1.5 m. The observation of the crowns was carried out by high-speed cameras. The static and dynamic parameters of crown formation were analysed. It was found that the crowns formed on the water film covering the saturated soil surface were smaller and the time intervals of their existence were shorter. In addition, the shapes of the crowns were different from those created on the water layer covering the glass surface. These differences can be explained by the slightly different values of surface tension and viscosity of the soil solution, the greater roughness of the soil surface and the lower thickness of the water film on the soil surface.

## Introduction

Soil, as the top layer of the Earth’s crust, undergoes continuous degradation induced by a variety of different factors [1,2]. This can be divided into chemical [3–6], biological [7–9] and physical degradation [10,11]. With respect to physical degradation, the main reported factors are: compactness [12,13], wind [14,15] and water erosion [16–18]. However, it must be stated that “water erosion” is a general term which covers a broad spectrum of processes: from the splash [19] through the movement of the soil and water mixture on the soil surface [20,21] to soil loss in streams and rivers [22]. Regardless of which form of erosion occurs, this phenomenon is unfavourable [23,24]. Therefore, it must be recognized and deeply understood in order to develop methods of soil protection.

The first stage of the water erosion process is the splash phenomenon. This phenomenon has been investigated by different researchers [25–27]. The method used most frequently for these investigations is the measurement of soil mass transfer [28,29]. However, new techniques may also be found, such as analysis of the splash particle traces [30], laser diffraction to measure the aggregate’s size after splash [31] the force transducer to measure the kinetic energy of drop impact [32,33], measurements of the sound wave energy [34] and high-speed cameras [26,35–37]. This last method (i.e., high-speed cameras) has become more popular in the last few years. It is not surprising, because those cameras allow for the splash to be recorded and then for the analysis of each stage from all interesting aspects. One of these aspects is crown formation after the water drop impact.

The issue of crown formation was investigated in several works [38,39]. The majority of them were carried out in liquid systems–where the water drop hits the water surface. The first study of this is probably the work of Worthington [40], in which the author investigated splash behaviour due to milk drop impacts from different heights. It is worth noting that this paper was published at the end of 19^th^ century. Great respect is due to the author that (at the then level of technology) he was able to carry out such detailed investigations. Amongst newer works, Cai et al. [41] can be mentioned, who investigated differences in liquid crown formation and droplet penetration during impact from very low heights onto a liquid surface. Manzello and Yang [42] investigated and compared water droplets impinging on water and HFE7100 (methoxyperfluorobutane 99%) surfaces with fluid depth varying from 2 mm to 25 mm. Vander Wal and Berger [43] observed splash phenomenon and crown behaviour using droplets of different fluids on the various depths of film of the same fluids. Kubota and Mochizuki [44] investigated the influence of the head shape of a solid body thrown into water (from a depth of more than 30 cm) on splash formation and crown type.

At the end of the previous century, there were published papers describing the crown formation on the wetted solid surface. Yarin and Weiss [45] investigated the term of splash/deposition limit and defined splashing threshold. Cossali et al. [46] tested the splash/crown formation on the thin liquid layer of water or water–glycerol mixtures on the surface of an aluminium disc. The crown formation recorded at sufficiently high-impact velocities on a thin film of liquid using silicon oils and water–glycerol mixture was observed by Rioboo et al. [47]. The effect on crown formation during oblique drop impact on a wet wall was used by Cheng and Lou [38]. Krechetnikov and Homsy [48] were trying to explain the crown-forming instability problem during water or milk drop splash on a thin film of the same liquid.

There are also some works which refer to other aspects related to the crown formation phenomenon. Some authors investigated crown behaviour in simultaneous drop impacts: Roisman et al. observed multiple drops impacting on a dry surface [49] and Raman et al. studied crown–crown interaction when two drops impacted onto a liquid film [39]. Zhang et al. [50] investigated the spectrum of small-amplitude perturbations growing on the crown rim during splash on thin liquid layer.

Summarizing the abovementioned articles, it can be stated that the crown formation process is related to some factors which can be divided into four groups: i) liquid properties (density, viscosity, viscoelasticity, surface tension etc.); ii) the energy of the drop; iii) water drop size (the diameter and for bigger sizes the drop shape); iv) the properties of the surface the drop hits. When this surface is of another liquid, the same types of properties (density, viscosity, viscoelasticity, surface tension etc.) as of the liquid from which the drop is formed are taken into account and, additionally, the depth of the liquid’s layer. When this surface is solid, the following quantities are investigated, among others: type of the solid phase, roughness or wettability [51]. In the case of water drops impacting on a soil surface, it is necessary to take into account soil texture, grain shape and size, and packing density [37].

The review of the literature was the impulse necessary to make the next step in understanding the course of the splash phenomenon on thin layers of liquids. As our investigations concern the splash erosion, in this work we focused on the surface of the saturated soil. Our preliminary test allowed us to formulate the following hypothesis: the splash phenomenon and the crown formed as a result of it will occur differently when the water drop falls on the thin water layer formed on a smooth, model surface than when it falls on the saturated soil surface. The aim of this study was to quantitatively describe these differences using the crown formation parameters.

## Materials and methods

The following conventions were adopted in this paper: i) the term *drop* means the water which falls on the different type of surface and causes the splash; ii) the term *droplet* means the water (often with soil particles) which is detached from the crown as the result of the splash. Sometimes the droplets are called secondary drops [38,46] or secondary droplets [47,51,52].

Measurements of the splash phenomenon were conducted on the saturated soil samples and thin water film on model, smooth surface.

### The thin water layer on the model, smooth surface

The thin water layer was created on a dry and clean Petri dish glass surface (97 mm external diameter) by dropping nine water drops and the crown was recorded for the tenth. Because of surface tension, the collected water created a circle with a diameter of about 5–6 mm. Such a procedure allowed us to obtain a reproducible thin (about 0.4 mm) water layer. The depth of this layer was the smallest (we were not able to decrease it) in the assumed temperature conditions because it was the resultant of the hydrophobicity of the surface and surface tension of water. The thin water layer placed on the smooth surface will hereafter be called the “water layer”.

Only one water drop was dropped onto such a layer, the crown formation was recorded and then the dish was carefully wiped and dried. For the next water drop, a new water layer was prepared. The measurements were made over 10 replications.

### The thin water layer on the saturated soil surface

Fluvic Endogleyic Cambisol (siltic) soil, taken in the north Poland (Janowka; 54°03N, 19°07’E) from the top layer (5–15 cm) was investigated. The authors declare that no specific permissions were required for these locations and confirm that the field studies did not involve endangered or protected species. According to the particle size distribution, this soil can be classified as silt loam (sand– 23.10%, silt– 67.06% and clay– 9.83%). The measurements were carried out over three replications using a laser diffractometer Mastersizer 2000 (Malvern, UK) with Hydro G dispersion units [53]. The contact angle of the investigated soil, measured by KRŰSS DSA 100 (Germany), was 32.3°(SD = 0.99).

Air dry soil samples were sieved through a 2 mm mesh, moisturized by mixing with distilled water and placed in the aluminium rings with 40 mm diameter and height of 10 mm. The rings were secured from the bottom by the chiffon in order to prevent soil loss. Then the rings were set to the vessel with the water to obtain the full saturation of the soil sample by capillary transport. Such a procedure allowed all the capillaries to be filled by water. However, there was not a water layer on the soil surface. The occurrence of the water layer on the surface is the prerequisite for obtaining the crown after the water drop hits the surface. To obtain the reproducible water layer, similar to above, nine water drops (one after another) were dropped onto the surface, and the crown was recorded for the tenth. The measurements were made over 10 replications (i.e., 10 cylinders were used).

### Water drop

The drops of water were created using a system consisting of a peristaltic pump, Aqua-trend Series 100 Micro (Aqua-trend, Poland), with a controller dosing distilled water with a flow rate of 9.6∙10^−7^ m^3^∙min^-1^. The temperature of water and in the room was 20°C ± 1°C. The glass capillary was used for the formation of the drops. It allowed us to obtain drops with a diameter of 4.2 mm (SD = 0.02 mm). The drops had been falling freely from a height of 1.5 m.

### Drop impact characteristics

The work of Yarin [51] contains a summary of the set of dimensionless numbers used to characterize the conditions during liquid drop impact on the thin layer of the same or other liquid. These are: the Weber number (We = *ς∙*D*∙*V^2^/σ), the Reynolds number (Re = *ς∙*D*∙*V/μ), the Ohnesorge number (Oh = μ/(*ς∙*σ*∙*D)^1/2^), the splashing parameter (K = We·Oh^-2/5^) and the dimensionless film thickness (H = h/D) (where D is drop diameter, V–drop impact velocity, *ς*, μ, σ –liquid density, viscosity and surface tension, and h–thickness of the pre-existing liquid film).

Based on the formulas presented above, we calculated those numbers for the water drop impact (with D = 4.2 mm; *ς =* 998.2 kg·m^-3^; μ = 0.001005 N·s·m^-2^; σ = 0.0725 N·m^-1^) released from 1.5 m height onto a water layer (0.4 mm) created on a glass dish surface: We– 1388; Re– 20441; Oh– 0.002; K– 17307 and H– 0.1.

The attempt to determine the same set of the numbers for the impact of the water drop onto a liquid layer created on the soil surface turned out to be difficult. The reason for this difficulty was the inability to estimate the viscosity and surface tension of the soil and water mixture created during consecutive drop impacts and the thickness of the film on the soil surface. The last problem was exacerbated by the small crater formation on the soil surface after the impact of the subsequent drops.

### System for recording the movies

Measurements were recorded using a high-speed camera (Vision Research Miro M310) with the speed of 3260 frames per second at the highest available resolution (1280 x 800 pixels) and Phantom Camera Control software. The camera was placed at a distance of 0.9 m. The optical axis of the camera was at the same level as the surface of the water layer (both for the rings with the soil and the Petri dishes).

For proper lighting of the samples, three LED panels (back lighting) were used, with dimensions of 0.6 m x 0.6 m and each of them guaranteed luminous flux of approximately 3500 lumens.

### Image analysis

The analysis of recorded material was based on pixels calculations for every image using Vision Assistant software (National Instruments). Because it was not always possible to place the ring with the sample perfectly in the same place, the actual size of the pixel could vary, so for all measurements the calibration of the pixel size was carried out. All photos were calibrated by comparing the size of the ring with the soil from the image with the actual size of the aluminium ring.

The first five consecutive frames were analysed for every image in order to investigate the dynamics of crown rising. This choice was a result of initial observations which showed that after this time (between the fifth and tenth frames) the crown on the soil began to break up. Counting of the frames started from the moment when the water drop made contact with the soil surface or water film. Based on the time interval between the next frames (3.06 ∙ 10^−4^ s), this analysed part of the splash phenomenon took 1.53 ∙ 10^−3^ s for every sample. In addition, the frame with the maximal value of the spread parameter of the crown (S) was analysed and, based on this frame, the maximal values for other parameters were calculated.

### Crown parameterization

In order to describe the differences between the crown formation on the investigated surfaces, two different times intervals were used:

- time of crown growth up (t_g_)–the maximal time interval counted from the moment of drop impact to the start of the breaking up of the crown (soil surface) or to the start of the crown collapsing (water layer, where the crown does not break up), expressed in ms,
- time of crown breaking up (t_b_)–the maximal time interval between t_g_ and complete disappearance of the crown, expressed in ms.

It should be noted that the sum of both time parameters gives the total duration of the crown phenomenon.

**Fig 1:**
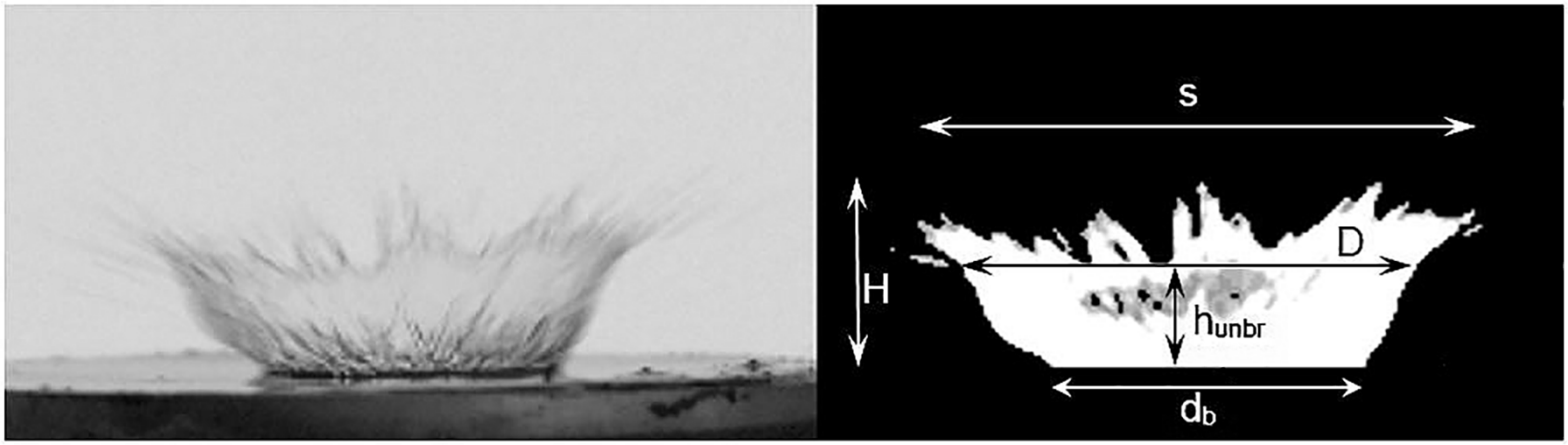
The static parameters of the crown. S–spread, H–height, D–diameter, h_unbr_−height of unbroken part of crown, d_b_−base diameter.

In addition to the parameters relating to the duration of the phenomenon, the following static parameters were analysed:

- the linear spread measured at the top of the crown, expressed in mm–S in Fig 1,
- the height of the crown (from base to the highest point), expressed in mm–H in Fig 1,
- the diameter of the crown (measured below the broken part of the crown or the crown rim), expressed in mm–D in Fig 1,
- the height of the unbroken part of the crown, expressed in mm–h_unbr_ in Fig 1,
- the base diameter of the crown (measured at the base of the crown), expressed in mm– d_b_ in Fig 1,
- the ratio of H and h_unbr_,

and two dynamic parameters:

- the velocity of a crown rising–vertical velocity based on the difference between crown height parameter values measured between two successive frames (i.e., in the first 1.53 ∙ 10^−3^ μs, between first and second frames, second and third etc. up to fifth frame), expressed in m∙s^-1^,
- the velocity of a crown spreading–horizontal velocity based on the difference of crown spread parameter values measured between two successive frames (i.e., in the first 1.53 ∙ 10^−3^ μs, between first and second frames, second and third etc. up to fifth frame), expressed in m∙s^-1^.

Some of above parameters were previously used by different authors. Cossali et al. [54] presented two kinds of crown diameter: the upper external diameter D_eu_ measured at the base of the rim (top of the crown) and the lower external diameter D_el_ measured at the crown base. The first one could be comparable with the diameter of a crown measured below the breakdown spot or crown’s rim presented in this study (D). The height of the unbroken part of the crown (h_unbr_) presented here could be related with the crown height parameter shown by Coghe et al. [54] and Cossali et al. [55].

### Statistical analysis

The obtained results were subjected to analysis to find the statistical significance of differences between the crowns on the thin layer on the soil surface and the layer on the smooth surface, taking into account all the presented parameters at different times (frames). The analysis was based on a Student’s t-test for independent samples at significance level α = 0.05.

## Results

### The shape of the crowns

**Fig 2:**
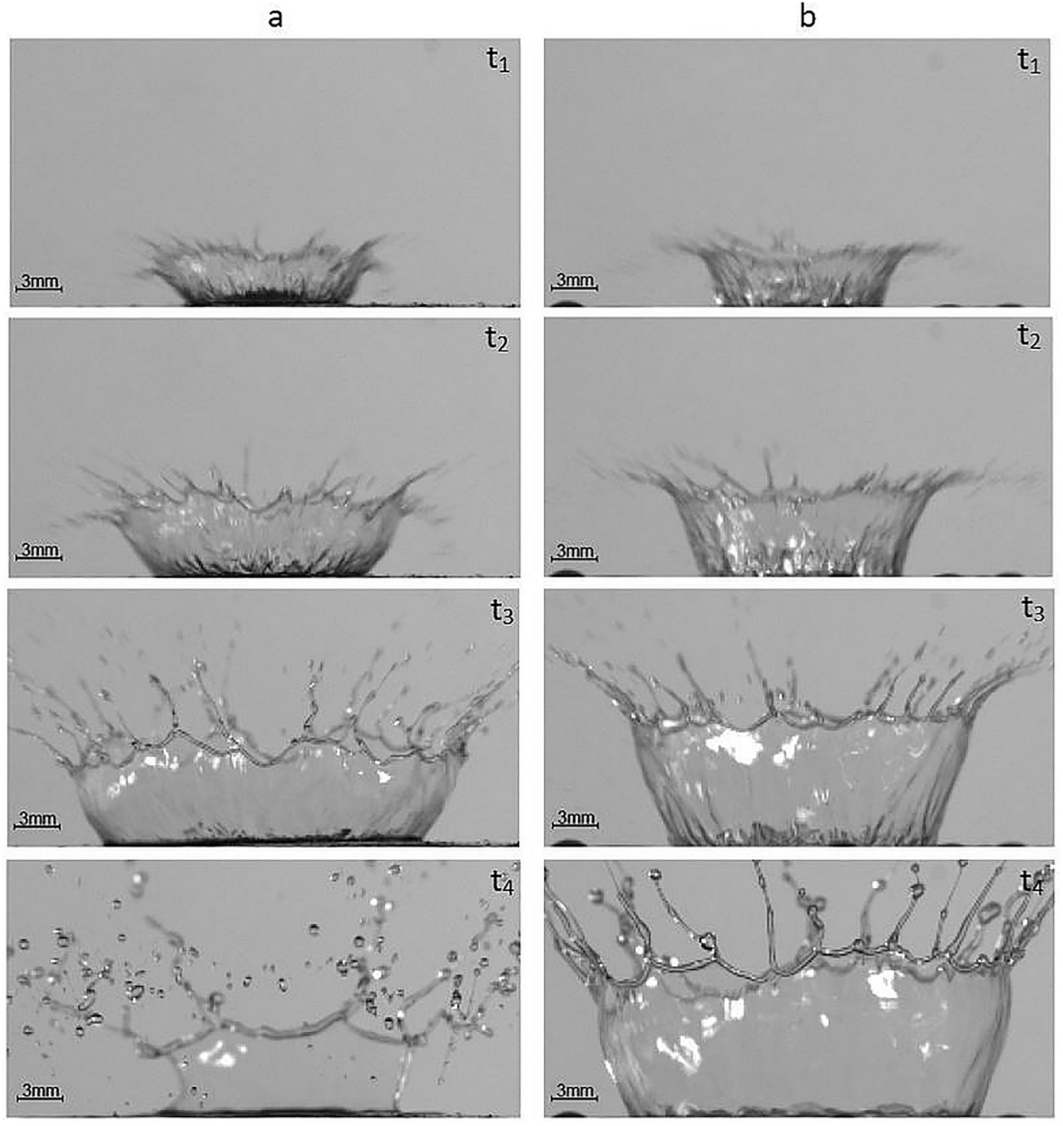
Comparison of crown shapes at different time intervals. (t_1_ = 0.92 ms, t_2_ = 1.53 ms, t_3_ = 3.06 ms, t_4_ = 7.65 ms) Time intervals after the drop impact from 1.5 m height for: (a) crown on soil surface; (b) crown on water layer.

The differences between crown shape on the soil surface and on the water layer in the same time intervals are shown in Fig 2.

It can be seen from the Fig 2 that the crown which was created on the soil surface disrupted and ended earlier. In addition, the sizes and shapes were different. The measures of these differences are expressed in the values of the investigated parameters.

### Time of crown’s growth up and breaking up

**Fig 3:**
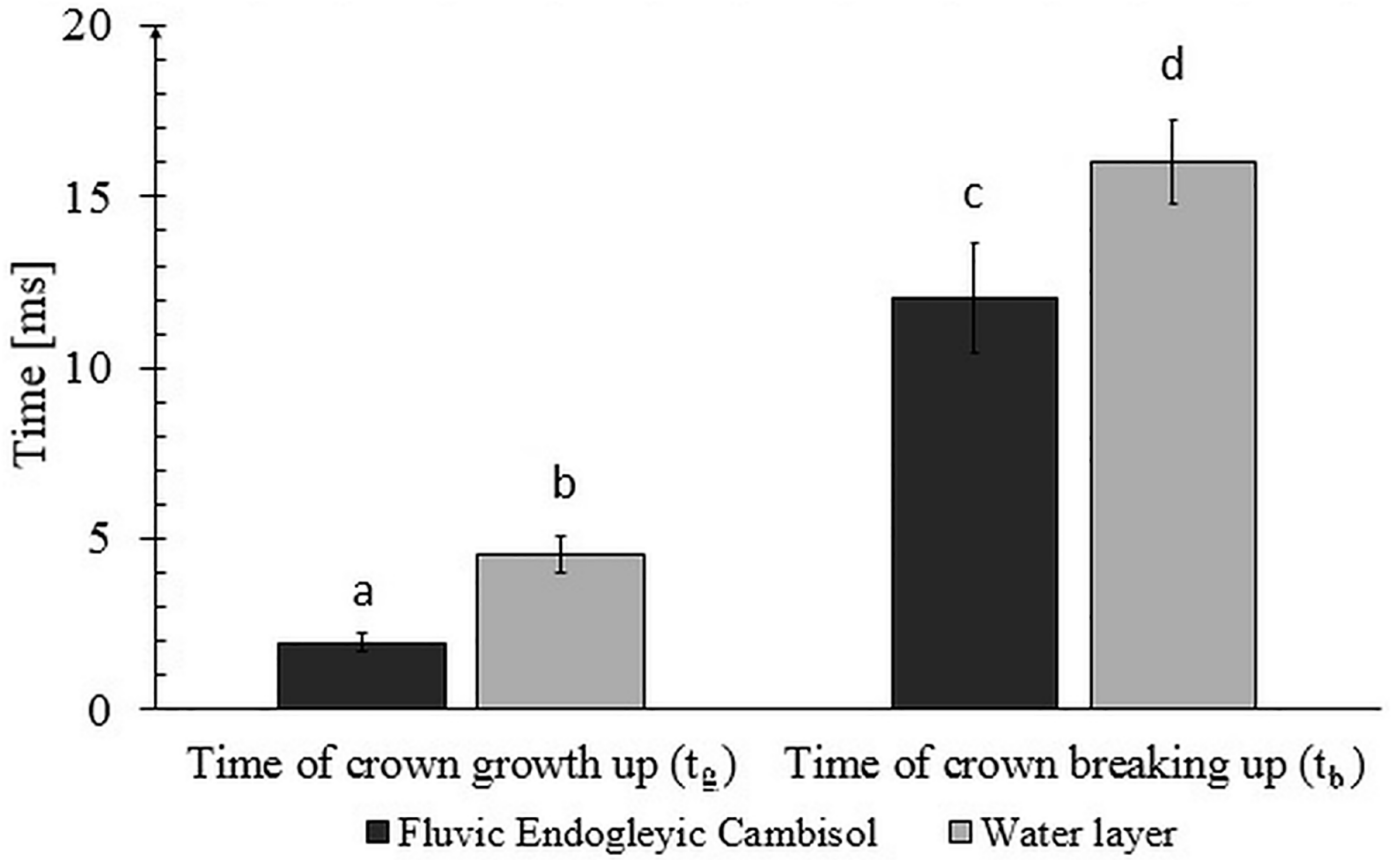
Times of crown’s growth up and breaking up on the saturated soil surface and water layer on the smooth surface.

The summary of measured time values t_g_ and t_b_ is presented in Fig 3 and S1 Table. It can be seen from this chart that the crown growth rates (both t_g_ and t_b_) were much bigger on the water layer surface. In consequence, the total duration (as mentioned in the *Materials and Methods* section, total duration is the sum of t_g_ and t_b_) was higher for the water layer than for the soil surface (20.58 ms and 14.01 ms respectively).

The second observation from Fig 3 is the fact that the duration of the crown breaking up was much longer than of the crown growth. This was observed for both investigated cases. However, there were the differences between them. This difference can be expressed by t_b_/t_g_−this ratio was about six times for the saturated soil surface while for water layer surface it was about four times.

### The static parameters

**Fig 4:**
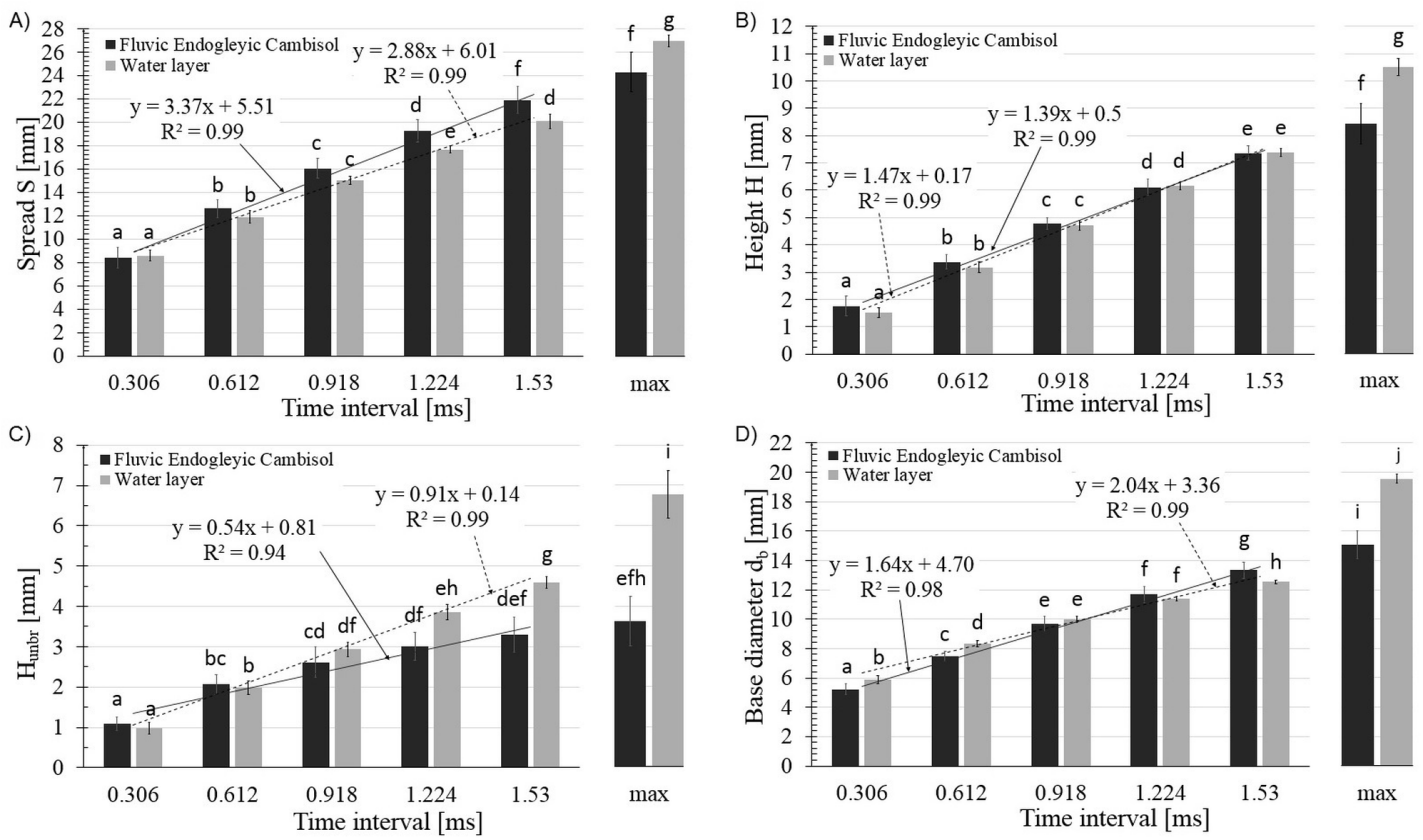
The static parameters of the crowns recorded in the first five subsequent frames and their maximal value recorded after the water drop hitting on the saturated soil surface and water layer on the glass surface. (A) the linear crown’s spread (S); (B) the crown’s height (H); (C) the height of the unbroken part of the crown (h_unbr_); (D) the crown’s base diameter (d_b_).

The values of static parameters recorded for the first five movie frames and maximal values of these parameters for the soil surface and water layer are presented in Fig 4 and S2 Table.

The analysis of data presented in Fig 4 (from A to D) leads to two general observations. The first is the statement that the differences were not visible in the initial phase of the course of the phenomenon (up to 1 ms). After the time interval equal to 1.224 ms, the course of the phenomenon started to vary (Fig 4A and 4C), with statistically significant differences obtained in all cases for the maximal values of the investigated quantities. Secondly, the increase of all investigated parameters was linear–as evidenced by the high value of the coefficient of determination R^2^.

Another static parameter of the risen crown was the crown’s diameter (D). This parameter took on a slightly lower value than the spread of crown (Fig 4A) and the values were statistically different only for the fifth frame and maximal value, which ranged from 18.8 mm (SD = 2.6 mm) to 25.6 mm (SD = 0.9) for the saturated soil surface and water layer respectively.

**Fig 5:**
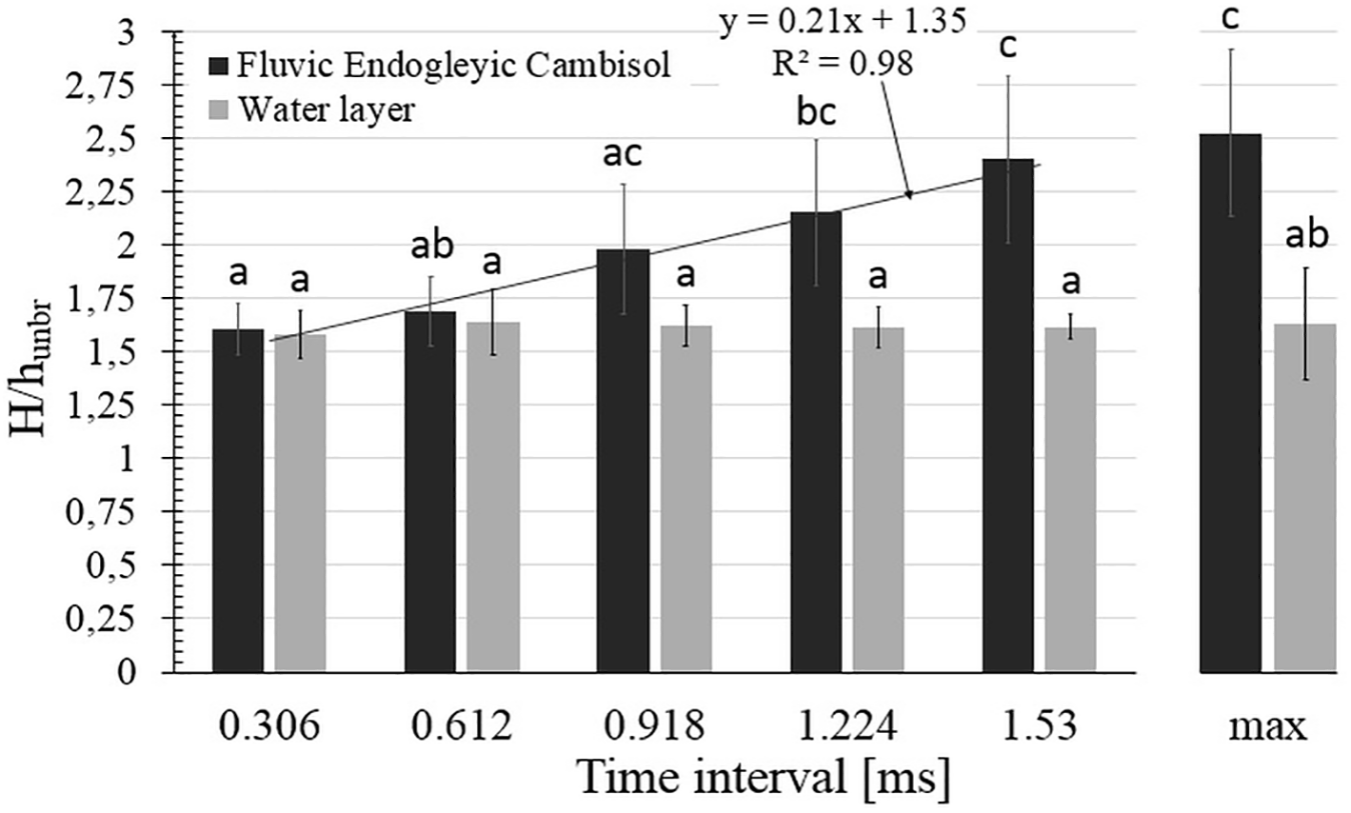
The ratio of the height and unbroken height of the crown (H/h_unbr_). Recorded in the first five subsequent frames (and its maximal value) after the water drop hitting the saturated soil surface and water layer on the glass surface.

Based on the values presented in Fig 4B and 4C, the ratio of H and h_unbr_ was calculated, with the results obtained shown in Fig 5 and S3 Table.

For the water layer, the ratio of H and h_unbr_ was stable for all five measured time intervals. The value of this ratio was also the same for the maximal size of the crowns. For the soil surface, this ratio increased, which means that the crown structure is much more irregular.

### The dynamic parameters

**Fig 6:**
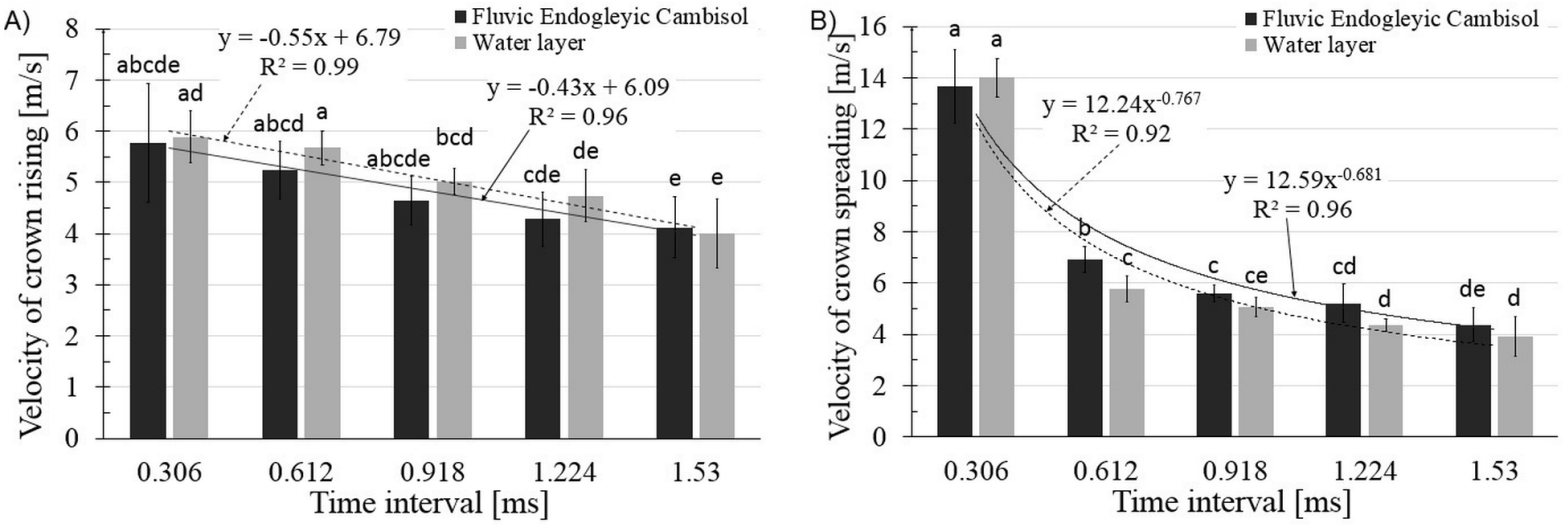
**The velocities of (A) crown rising (vertical velocity) and (B) crown spreading (horizontal velocity) for the saturated soil and water layer**.

The values of the investigated dynamic parameters are shown in Fig 6 and S4 Table. The first general observation is that in all cases the differences between both surfaces, within the same time intervals, were not statistically significant. However, it can be seen that generally for crown rising the vertical speed and for crown spreading the horizontal speed were slightly higher for the water layer.

Secondly, the shapes of the presented dependencies were different. The highest velocities were observed in the first moments after the water drop’s impact and then the velocities decreased. For the crown rising (Fig 6A), the linear interpolation was more suitable. For the crown spreading (Fig 6B), the exponential curve was better. However, taking into account the essence of the phenomenon, the exponential interpolation was used in both cases. It just seems that the time interval for the crown rising was so short that the plateau has not yet occurred. However, the plateau should be expected because the crown rising is, of course, limited. The crown spreading rate was the highest in the first frame immediately after the drop impact and its value was 13.7 m ∙ s^-1^ for the saturated soil surface and 14 m ∙ s^-1^ for the water layer. In the subsequent phase of the crown rising, the velocity was significantly reduced (by about 50–60%) for both cases and values were 6.9 m ∙ s^-1^ for the soil sample crown and 5.76 m ∙ s^-1^ for the water layer. It should be noted that by analysing both velocity plots in the initial stage of crown formation, the velocity of spreading (horizontal velocity) is more than double compared with the rising velocity (vertical velocity).

## Discussion

It can be stated that the values of the static parameters were very similar in the early stages of the crown rising and started to vary in the later stages, especially in the time intervals corresponding to the maximal values of each parameter. Generally, the maximal values showed that the crowns created on the saturated soil surface had smaller dimensions compared with the crowns created on the water layer covering the model, glass surface. They were narrower (Fig 4A and 4D) and smaller (Fig 4B and 4C). Comparing the dynamic parameters, it should be stated that the crowns on the soil surface lasted a significantly shorter time (Fig 3). The question arises: what were the reasons for such differences?

To begin with, we will identify which factors influence the crown formation phenomenon. We will consider them all separately and then discuss their complex effects.

### The issue of liquid film thickness

The thickness of the liquid layer on the surface influences the course of the splash phenomenon [54,56]. In the case of our experiment, it seems to be one of the most important factors. The greater the thickness of the liquid film, the greater the shielding effect of the surface roughness. However, it is impossible to estimate to what extent the liquid film thicknesses differed in both systems. While it is still possible to roughly determine the thickness of the water layer on the glass surface (about 0.4 mm), we are not able to specify it on the saturated soil surface. Furthermore, this thickness could be different from splash to splash. This was because the water from previous drops that hit the surface then created a thin layer on surface and slowly infiltrated into the bulk of the soil. However, because of the surface roughness, and soil porosity and heterogeneity, the efficiency of such infiltration was different.

It was possible to eliminate the effect of the changing of the soil properties after the successive drop impacts. We could just add appropriate amount of water directly on the surface–not as the hitting water drops. However we rejected such procedure. What arguments were in favor of this rejection? Adding gently the water directly onto the soil surface we would keep the surface intact i.e. without the creation of the crater. However this situation will last only to the moment of the water drop hitting. The first water drop would create not only the crown but also the crater. Thus the energy of the water drop would be divided into two sub-processes. Therefore we (being aware that this is also the source of the error in the interpretation of the results) wanted to have the situation when the crater was formed by the previous water drops (the crater is not significantly increase after some impacts) and the energy of measured water drop was used only onto the crown formation. According to us, such procedure would ensure more repeatable conditions than in the case when crater was created. And additionally this system is closer to the real situation in the nature (a splash during the rain).

Regardless of how a layer of water was formed on the surface of saturated soil we were not able to measure the water layer thickness and we could only speculate (on the basis of our qualitative observations) that the thickness of the liquid film was smaller on the soil surface than on the glass. If so, the effect of the surface roughness could be significant and as the result it could influence the dimensions of the crowns, their shapes and their regularity.

### The issue of surface roughness

Another very important factor which could influence the differences between both systems was the surface roughness. The glass surface was smooth while the soil surface had microrelief [43]. Taking this into account, it can be expected that the crown created on the water layer on the glass surface should be more reproducible than one on the soil surface. We consciously use the term reproducible (and repeatable) [57]. This is because it is not possible to obtain the same repeatable splash phenomenon. The shape, size, speed and rotation of the water drop differs for the subsequent drops and the place the water drop hits is also not the same–this last is the most important factor when the surface is not homogeneous. When such a situation occurs in the case of a rough soil surface, it can be expected that the roughness of the soil surface will influence the decrease in the crown dimensions because some of the energy after the splash is needed to overcome the resistance on the rough surface [43]. Taking into account the results presented in photos by Vander Wal et al. [58], we could also state that the roughness of the surface has a considerable influence on the crown breaking up, especially in our work in the case of the saturated soil surface, where even a single particle of soil could disturb the rising of the crown wall and lead to an irregular structure.

### The issue of surface tension

In the case of the phenomenon on the water layer on the glass surface, the properties of the water did not change and it was still the same water as before the splash. The situation was different in the case of the splash on the soil surface because of the contact with the soil and soil solution which covered the surface. The crown and droplets were formed by the mixture of the pure water (from the drop), soil solution and detached soil particles–this mixture hereafter will be referred to as “splashed mixture”. As it is impossible to specify the mixing efficiency, it should be assumed that the splashed mixture was not homogeneous throughout the whole volume.

The mixing of water from the drop with the soil solution could lead to a higher concentration of ions in the splashed mixture compared with the pure water that was splashed in the case of the water layer covering the glass surface. The higher concentration of ions causes the higher value of surface tension [59]. In turn, the higher surface tension causes the smaller sizes of the crown (height), which is consistent with the results obtained by Liang et al. [56].

The increase in the concentrations of ions was not the only factor which caused the change of the surface tension in the splashed mixture. The second factor could be the detached soil particles. According to the literature, the appearance of the silt particles first causes the decrease in the surface tension–to the minimum. The increase in the concentration of the particles in the suspension results in the following increase in surface tension, up to the value similar to that of pure water [60,61].

We are not able to estimate quantitatively to what extent and in which proportions the above-specified two opposite phenomena took place in our experiments. However, on the basis of the qualitative observations, we can speculate that the more significant effect was caused by the increase in the concentrations of the ions. In other words, we had the resultant slight increase in surface tension (and, consequently, the lower We number) compared with pure water. The argument for this statement is the following: we were able to assess visually that a very small number of soil particles were detached from the surface and transported in the crown structure. Simply speaking, the crown wall was transparent and there were only single traces on the paper on which the splashed droplets had fallen.

There is also a second argument. The measure of surface tension is the energy necessary to increase the surface of the liquid. The growth of the crown is just the pure realization of such a definition–the increase in the crown is the increase in the surface of the liquid. The bigger the surface tension, the bigger the counterforce to increase the crown’s dimensions. This seems to be one of the factors influencing the smaller dimensions of the maximum crown parameters (Fig 4).

### The issue of viscosity

The appearance of the ions and solid particles in the suspension which created the crown and droplets when the water drop hit the saturated soil surface changed not only the surface tension but also the viscosity. It can be found in the literature that the viscosity of the solutions with ions is higher than the viscosity of the water [62]. In addition, the presence of solid phase particles could increase the viscosity [63]. If so, we can conclude that the suspension which created the crown and the droplets after the splash on the saturated soil surface has slightly higher viscosity than the pure water after the splash on the water layer covering the glass.

### The issue of density

The mixing of the water with soil particles reliably increases the density of the suspension and as the result it can be expected that the crown height would be smaller. However, it should be stated that in the splash the soil particles detached from the surface did not create the homogeneous suspension. It means that the amount (concentration) of the solid phase is different in different areas of the crown. If so, it seems that it is not justified to conclude that this factor influences the crown height. It should rather be stated that the presence of soil particles influences the diversity in crown height at different points on the perimeter.

### The issue of the influence of the complex factors

Each of the above mentioned factors could have an impact on the splash. It is difficult to assess which factors and to what extent, but it can be expected that for thin layers of water the influence of the these water depths and surface roughness is much greater than changes of surface tension and viscosity. Regardless of which factor had the most impact all of them should be considered together.

Because there is a lack of papers describing the factors influencing the crown formation on the saturated soil surface, we had to discuss the works in which other systems were investigated and then, while being aware of the differences, we tried to conclude in relation to our study.

The question arises: which factor (surface tension or viscosity, or maybe roughness of surface and thickness of liquid layer) had greater impact on the resultant effect? Vander Wal et al. [43] investigated the crown formation in eight liquids which had different properties–including surface tension, viscosity and density. The cited authors stated that the viscosity could have greater influence than surface tension, because besides the effects on splashing droplets, it could also have some effect on the thickness of the crown wall and uniformity of the crown structure. This conclusion may be true in pure liquid systems. However, it is difficult to transfer this statement directly to the system using a liquid suspension or solid surface.

Some essential results can be found in the paper of Liang et al. [56]. The authors used physical and mathematical models to predict the crown formation in the liquids with different properties for different liquid thicknesses. They showed that the crown height increases with the increasing Weber number and that the crown diameter does not depend on this number. It means that, according to Liang’s model, the crown height decreases with the increase in surface tension. Taking into account the maximal values of crowns height in our work (Fig 4B and 4C), we could state that a slight increase in surface tension in the case of the soil saturated surface could influence this static parameter and result in a lower crown.

The same authors concluded that the Reynolds number did not influence any crown dimensions–neither diameter nor height [56]. In other words, they suggested that the crown dimensions did not depend on the liquid density and viscosity, or that the influence of each factor cancels out the influence of the other. It is difficult to interpret such results unambiguously in the light of our results. It must be stated that the results of the simulations did not concern the specific substances but only the hypothetical liquids–i.e., the values of investigated properties had been changing in the assumed ranges. Moreover, although our value for the Weber number was included in the range investigated by Liang et al. [56], the value of Reynolds number was outside this range (their range varied between 1,168 and 13,676 and we had Re equal to 20,441). Besides this, it should be remembered that the different situation could have arisen because of the changes in surface tension and viscosity due to differences between the properties of liquids and those of solid particles in suspension.

Additionally, the other aspects (liquid film thickness and surface roughness) should be taken into account. Liang et al. [56] found that the crown diameter can be increased by decreasing the film thickness, while in the case of the crown height the effect is more complicated. In turn, Cossali et al. [54] stated that the crowns growing velocities depend slightly on the film thickness.

Considering all of presented factors, we could state that the crown formation on the saturated soil surface was slightly affected by the increase in surface tension and viscosity due to the appearance of the ions and solid particles in the suspension, which created the crown. In addition, the thickness of the liquid film (suspension on mixture of water and soil) covering the soil surface and roughness of the soil surface had an influence on the static parameters of the crowns.

### The issue of the velocity of the crown formation

The static parameters which were discussed in the previous sections concerned the crowns’ dimensions up to their maximal values. However, as it can be seen from Fig 3, the time intervals in which the crowns reached the maximum sizes were different for both systems (the differences were statistically significant)–the crown formation lasted much longer when the crown was created on the water layer on the glass surface (total duration of crown phenomenon 20.58 ms). At the same time, the analysis of data presented in Fig 6 showed that the dynamics of the crown rising in the first moments after the drop impact were practically the same (the differences between water layer on the glass and soil surfaces were not statistically significant). It follows from this that the differences arise later.

It seems that the explanation of such dependences can be based on factors similar to those used in the crown dimension analysis–i.e., surface tension, viscosity and surface roughness. However, their impact becomes apparent only after some time. In the first moments after the water drop hitting, the factor which most influenced the phenomenon of the crown formation was the kinetic energy of the falling drop. This energy was the same for both systems–hence the lack of statistical differences between systems in Fig 6. However, when the crowns started to be bigger, the bigger values of surface tension, viscosity and roughness of the soil surface simultaneously counteracted the growth of the crown. This resulted not only in dimensions of the crown but also in velocity of crown rising and spreading.

## Conclusions

1. It was found that there were statistically significant differences between the crown formation on the thin liquid film covering the saturated soil surface and the water layer on the smooth glass surface. In the early stages of the crowns development, those differences were unnoticeable but started to vary in the later stages, especially in time intervals with maximal values of parameters. The crowns formed on the saturated soil surface were smaller (lower and narrower) and the time intervals of their existence were shorter. In addition, the shapes of the crowns were different from those created on the water layer covering the glass surface.
2. These differences can be explained by the combination of the following factors which differed between each system: i) the lower thickness of the liquid layer covering the soil surface; ii) the greater roughness of the soil surface compared with the smooth glass; iii) the slightly bigger value of surface tension in the soil solution covering the soil surface; iv) the slight increase in the viscosity of the splashed suspension; v) the increase in the density of the splashed suspension–mainly due to the presence of the solid soil particles. Two first specified factors seems to have the biggest impact on the differences of the crown formation on the thin water layer on the saturated soil surface and model, smooth glass surface.

## Supporting information

S1 Table Times of crown’s growth up and breaking up [ms] on the saturated soil surface and water layer on the smooth, model surface. The values are expressed in ms. SD–represents sample standard deviation of 10 repetitions.
(DOCX)

S2 Table The static parameters of the crowns recorded in the first five subsequent frames and their maximal value recorded after the water drop hitting on the saturated soil surface and water layer on the model surface. The values are expressed in mm. SD–represents sample standard deviation of 10 repetitions.
(DOCX)

S3 Table The ratio of the height and unbroken height of the crown (H/h_unbr_). SD–represents sample standard deviation of 10 repetitions.
(DOCX)

S4 Table The velocities of crown rising (vertical velocity) and crown spreading (horizontal velocity) for the saturated soil and water layer on model surface. The values are expressed in m/s. SD–represents sample standard deviation of 10 repetitions.
(DOCX)
