# Supplementary material for: The differences in crown formation during the splash on the thin water layers formed on the saturated soil surface and model surface
Source: PLoS One. 2017 Jul 27;12(7):e0181974. doi: 10.1371/journal.pone.0181974 (PMC5531603; doi:10.1371/journal.pone.0181974)
Supplement: S1 Table — The values are expressed in ms. SD–represents sample standard deviation of 10 repetitions. (DOCX) [file pone.0181974.s001.docx]

SUPPORTING TABLE S1 for

**The Differences in Crown Formation During the Splash on the Thin Water Layers Formed on the Saturated Soil Surface and Model Surface**

Michał Beczek, Magdalena Ryżak, Agata Sochan, Rafał Mazur, Cezary Polakowski, Andrzej Bieganowski

**S1 Table.** **Times of crown’s growth up and breaking up [ms] on the saturated soil surface and water layer on the smooth surface.**

| **Time of crown growth up (t_g_)** | | | |
| --- | --- | --- | --- |
| Fluvic Endogleyic Cambisol | 1/2*SD | Water layer (model surface) | 1/2*SD |
| 1.96 | 0.29 | 4.53 | 0.51 |
| **Time of crown breaking up (t_b_)** | | | |
| Fluvic Endogleyic Cambisol | 1/2*SD | Water layer (model surface) | 1/2*SD |
| 12.06 | 1.62 | 16.05 | 1.22 |
