# Supplementary material for: The differences in crown formation during the splash on the thin water layers formed on the saturated soil surface and model surface
Source: PLoS One. 2017 Jul 27;12(7):e0181974. doi: 10.1371/journal.pone.0181974 (PMC5531603; doi:10.1371/journal.pone.0181974)
Supplement: S2 Table — The values are expressed in mm. SD–represents sample standard deviation of 10 repetitions. (DOCX) [file pone.0181974.s002.docx]

SUPPORTING TABLE S2 for

**The Differences in Crown Formation During the Splash on the Thin Water Layers Formed on the Saturated Soil Surface and Model Surface**

Michał Beczek, Magdalena Ryżak, Agata Sochan, Rafał Mazur, Cezary Polakowski, Andrzej Bieganowski

**S2 Table.** **The static parameters of the crowns [mm] recorded in the first five subsequent frames and their maximal value recorded after the water drop hitting on the saturated soil surface and water layer on the glass surface.**

| A) | **The linear crown’s spread (S)** | | | |
| --- | --- | --- | --- | --- |
| Time interval [ms] | Fluvic Endogleyic Cambisol | 1/2*SD | Water layer (model surface) | 1/2*SD |
| 0.306 | 8.37 | 0.89 | 8.57 | 0.45 |
| 0.612 | 12.60 | 0.78 | 11.90 | 0.53 |
| 0.918 | 16.03 | 0.84 | 15.00 | 0.36 |
| 1.224 | 19.22 | 0.96 | 17.67 | 0.30 |
| 1.53 | 21.91 | 1.13 | 20.07 | 0.63 |
| max | 24.27 | 1.70 | 26.95 | 0.47 |
|  |  |  |  |  |
| B) | **The crown’s height (H)** | | | |
| Time interval [ms] | Fluvic Endogleyic Cambisol | 1/2*SD | Water layer (model surface) | 1/2*SD |
| 0.306 | 1.77 | 0.35 | 1.51 | 0.18 |
| 0.612 | 3.37 | 0.26 | 3.18 | 0.19 |
| 0.918 | 4.79 | 0.21 | 4.71 | 0.19 |
| 1.224 | 6.10 | 0.30 | 6.16 | 0.15 |
| 1.53 | 7.36 | 0.28 | 7.38 | 0.16 |
| max | 8.43 | 0.73 | 10.52 | 0.32 |
|  |  |  |  |  |
| C) | **The height of unbroken part of the crown (h_unbr_)** | | | |
| Time interval [ms] | Fluvic Endogleyic Cambisol | 1/2*SD | Water layer (model surface) | 1/2*SD |
| 0.306 | 1.09 | 0.17 | 0.98 | 0.15 |
| 0.612 | 2.07 | 0.25 | 1.98 | 0.17 |
| 0.918 | 2.61 | 0.38 | 2.94 | 0.18 |
| 1.224 | 3.01 | 0.34 | 3.86 | 0.19 |
| 1.53 | 3.29 | 0.43 | 4.59 | 0.15 |
| max | 3.62 | 0.62 | 6.77 | 0.59 |
|  |  |  |  |  |
| D) | **The crown’s base diameter (d_b_)** | | | |
| Time interval [ms] | Fluvic Endogleyic Cambisol | 1/2*SD | Water layer (model surface) | 1/2*SD |
| 0.306 | 5.23 | 0.36 | 5.87 | 0.27 |
| 0.612 | 7.48 | 0.34 | 8.31 | 0.22 |
| 0.918 | 9.67 | 0.50 | 9.97 | 0.20 |
| 1.224 | 11.69 | 0.55 | 11.39 | 0.17 |
| 1.53 | 13.33 | 0.55 | 12.54 | 0.12 |
| max | 15.06 | 0.95 | 19.57 | 0.30 |
