# Supplementary material for: The differences in crown formation during the splash on the thin water layers formed on the saturated soil surface and model surface
Source: PLoS One. 2017 Jul 27;12(7):e0181974. doi: 10.1371/journal.pone.0181974 (PMC5531603; doi:10.1371/journal.pone.0181974)
Supplement: S3 Table — SD–represents sample standard deviation of 10 repetitions. (DOCX) [file pone.0181974.s003.docx]

SUPPORTING TABLE S3 for

**The Differences in Crown Formation During the Splash on the Thin Water Layers Formed on the Saturated Soil Surface and Model Surface**

Michał Beczek, Magdalena Ryżak, Agata Sochan, Rafał Mazur, Cezary Polakowski, Andrzej Bieganowski

**S3 Table.** **The ratio of the height and unbroken height of the crown (H/h_unbr_)**

|  | **The ratio of H/h_unbr_** | | | |
| --- | --- | --- | --- | --- |
| Time interval [ms] | Fluvic Endogleyic Cambisol | 1/2*SD | Water layer (model surface) | 1/2*SD |
| 0.306 | 1.60 | 0.12 | 1.58 | 0.11 |
| 0.612 | 1.69 | 0.16 | 1.63 | 0.15 |
| 0.918 | 1.98 | 0.31 | 1.62 | 0.10 |
| 1.224 | 2.15 | 0.34 | 1.61 | 0.09 |
| 1.53 | 2.40 | 0.39 | 1.62 | 0.06 |
| max | 2.52 | 0.39 | 1.63 | 0.26 |
|  |  |  |  |  |
