# Supplementary material for: The differences in crown formation during the splash on the thin water layers formed on the saturated soil surface and model surface
Source: PLoS One. 2017 Jul 27;12(7):e0181974. doi: 10.1371/journal.pone.0181974 (PMC5531603; doi:10.1371/journal.pone.0181974)
Supplement: S4 Table — The values are expressed in m/s. SD–represents sample standard deviation of 10 repetitions. (DOCX) [file pone.0181974.s004.docx]

SUPPORTING TABLE S4 for

**The Differences in Crown Formation During the Splash on the Thin Water Layers Formed on the Saturated Soil Surface and Model Surface**

Michał Beczek, Magdalena Ryżak, Agata Sochan, Rafał Mazur, Cezary Polakowski, Andrzej Bieganowski

**S4 Table.** **The velocities [m/s] of crown rising (vertical velocity) and crown spreading (horizontal velocity) for the saturated soil and water layer.**

| A) | **Velocity of crown rising** | | | |
| --- | --- | --- | --- | --- |
| Time interval [ms] | Fluvic Endogleyic Cambisol | 1/2*SD | Water layer (model surface) | 1/2*SD |
| 0.306 | 5.77 | 1.16 | 5.89 | 0.51 |
| 0.612 | 5.25 | 0.56 | 5.68 | 0.33 |
| 0.918 | 4.65 | 0.48 | 5.02 | 0.26 |
| 1.224 | 4.28 | 0.52 | 4.74 | 0.51 |
| 1.53 | 4.11 | 0.60 | 3.99 | 0.68 |
| max | 3.13 | 0.58 | 0.78 | 0.07 |
|  |  |  |  |  |
| B) | **Velocity of crown spreading** | | | |
| Time interval [ms] | Fluvic Endogleyic Cambisol | 1/2*SD | Water layer (model surface) | 1/2*SD |
| 0.306 | 13.67 | 1.45 | 14.01 | 0.74 |
| 0.612 | 6.92 | 0.50 | 5.77 | 0.51 |
| 0.918 | 5.60 | 0.34 | 5.06 | 0.37 |
| 1.224 | 5.21 | 0.76 | 4.36 | 0.24 |
| 1.53 | 4.38 | 0.68 | 3.93 | 0.77 |
| max | 3.96 | 0.78 | 0.88 | 0.14 |
|  |  |  |  |  |
